# Characterization and Exploration of Placket–Burman-Designed Porous Calcium Carbonate (Vaterite) Microparticles

**DOI:** 10.1021/acsomega.3c05050

**Published:** 2023-11-15

**Authors:** Avi Singh, Sabya Sachi Das, Priya Ranjan Prasad Verma, Janne Ruokolainen, Kavindra Kumar Kesari, Sandeep Kumar Singh

**Affiliations:** † Department of Pharmaceutical Sciences and Technology, Birla Institute of Technology, Mesra, Ranchi, Jharkhand 835215, India; ‡ School of Pharmaceutical and Population Health Informatics, DIT University, Dehradun, Uttarakhand 248009, India; § Department of Applied Physics, School of Science, Aalto University, Espoo 00076, Finland; ∥ Research and Development Cell, Lovely Professional University, Phagwara, Punjab 144411, India

## Abstract

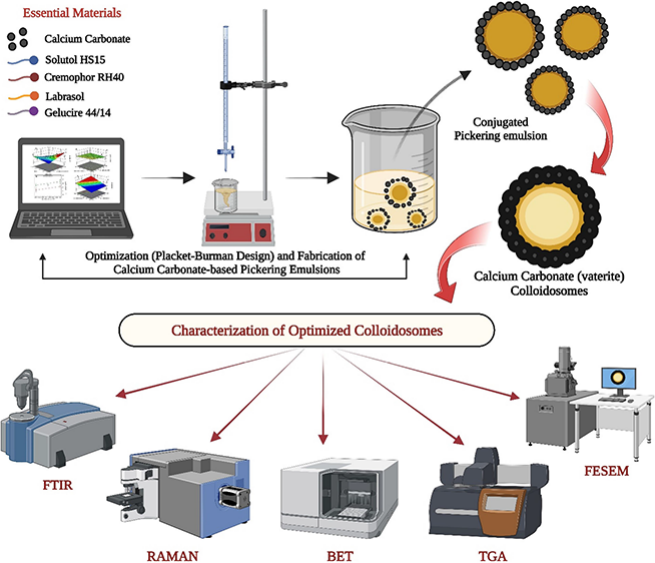

The objective of the research was to identify significant variables that impact the porosity-related properties of CaCO_3_ particles. The Placket–Burman design was employed to screen multiple variables, including pH, molar concentrations of calcium chloride and sodium carbonate, temperature, concentration of Gelucire 44/14, Cremophor RH40, Solutol HS15, Labrasol, mixing rate, reaction time, and order of addition. The response variables were surface area, pore radius, and pore volume. Influential methodologies such as XRD, FTIR, Raman spectroscopy, and TGA were utilized to validate the precipitate type. The BET surface area ranged from 1.5 to 16.14 m^2^/g, while the pore radius varied from 2.62 to 6.68 nm, and the pore volume exhibited a range of 2.43 to 37.97 cc/gm. Vaterite structures with spherical mesoporous characteristics were observed at high pH, whereas calcite formations occurred at low pH. The order of addition impacted the surface area but did not affect the pore volume. To maximize the surface area, a lower reaction time and molar concentrations of sodium carbonate were found to be advantageous. The pore radius was influenced by the pH, surfactants, and reaction conditions. The sediments were categorized based on the percentage of vaterite formation. The instrumental techniques effectively characterized the precipitates and provided a valuable complementary analysis.

## 1 Introduction

Porous vaterite calcium carbonate (CaCO_3_), owing to large porosity, high surface area, and rapid decomposition in acidic conditions, contributes to a practical and alternate drug delivery choice/sacrificial template to ferry drugs.^1,2^ Vaterite CaCO_3_ has been reported as a host for various moieties such as doxorubicin,^3^ antimicrobials,^4^ rhodamine B,^5^ photosens,^6^ and methotrexate,^7^ because of the inherently high surface area. The occurrence of vaterite in nature is relatively rare, primarily due to thermodynamic constraints that hinder the direct transformation of calcite into vaterite.^8^ Among various synthetic approaches (such as biomimetic synthesis and CO_2_-bubbling method) for preparing vaterite particles, the precipitation method using precursor salts (Na_2_CO_3_ and CaCl_2_) is relatively simple and industrially feasible.^8,9^ Mixing of the precursor salt seems to be an attractive alternative for the preparation of porous vaterite; however, the number of independent variables such as pH, temperature, reaction time, mixing mode, additives (surfactants, polymers, biomolecules, amino acids), and solvent ratio needs to be aptly controlled and optimized for its preparation. In the present research, we aim to screen and evaluate the critical independent variables (InV) influencing the response variables related to porosity such as surface area, pore radius, and pore volume. To achieve this, we employed the Placket–Burman design (PBD). The selected InV values were as follows: (a) pH, (b) molar concentration of calcium chloride, (c) molar concentration of sodium carbonate, (d) temperature, (e) concentration of additives (Gelucire 44/14, Cremophor RH40, Solutol HS15, Labrasol), (f) mixing rate, (g) reaction time, and (h) order of addition (L1, L2). Additives (Gelucire 44/14, Cremophor RH40, Solutol HS15, Labrasol) were selected based on our previous report that gave a maximum mole fraction of vaterite compared to calcite and aragonite.^5^

Considering the apparent relationship between drug loading, surface area, pore radius, and pore volume were selected as response variables (ReV). Among various screening designs, we have chosen the PBD because it can identify the main factors from a pool of many InV (as in the present work) that have the most impact on the selected responses.^10^ During our literature review on preparing porous vaterite CaCO_3_, we identified a gap in knowledge regarding the relationship between different variables and their impact on the surface area, pore radius, and pore volume. These properties are crucial in determining the drug loading capacity and release behavior of the bioactive substances. To address this gap, the present research focuses on screening and evaluating the variables influencing response (surface area, pore radius, and pore volume) to enhance our understanding of this field. In one of our previous studies, we studied the impact of eleven different excipients on the phase transition behaviors of calcite, vaterite, and aragonite.^5^ To ascertain the authenticity of the precipitates formed through the PBD, we validated them by employing X-ray diffraction (XRD), Fourier transform infrared (FTIR) spectroscopy, Raman spectroscopy (RS), and thermogravimetric analysis (TGA). Also, we have classified the precipitates into three distinct groups based on vaterite content. Group 1 consists of sediments with a vaterite percentage exceeding 75%; Group 2 includes residues with a vaterite content ranging from 45% to 70%; and Group 3 exclusively comprises calcite forms. This classification provides valuable insights into the varying compositions of the residues and facilitates a more comprehensive analysis of the experimental results.

## 2 Materials and Methodology

### 2.1 Materials

Calcium chloride (CaCl_2_) and sodium carbonate (Na_2_CO_3_) were acquired from Sigma Aldrich (India) for this study. Additionally, Solutol HS15 and Cremophor RH40 were generously provided as gift samples by BASF (USA), while Labrasol and Gelucire 44/14 were supplied ex gratia by Gattefosse (France). A mechanical stirrer (IKA RW 20 digital, India) was used to mix the contents. GPT-4 and Grammarly served as AI-assistants in drafting the final manuscript.

### 2.2 Synthesis of Calcium Carbonate Particle Using PBD

PBD is a fractional factorial design that uses a smaller number of runs than a complete factorial design while still being able to identify the main effects of the independent variables.^11^ A 16-run PBD was used to explore the eleven potential factors: pH (A: 4–12), the molar concentration of calcium chloride (B: 1.0–2.0 M), the molar concentration of sodium carbonate (C: 1.0–2.0 M), temperature (D: 4.0–60.0 °C), Gelucire 44/14 concentration (E: 1.0–4.0% w/v), Cremophor RH40 concentration (F: 1.0–4.0% w/v), Solutol HS15 concentration (G: 1.0–4.0% w/v), Labrasol concentration (H: 1.0–4.0% w/v), mixing rate (J: slow (2 mL/min) and instantaneous), reaction time (K: 0.08–10.0 h), and order of addition (L1: calcium chloride in the burette and sodium carbonate in a beaker; L2: sodium carbonate in the burette and calcium chloride in beaker) on response outcomes of surface area (Y1), pore radius (Y2), and pore volume (Y3). Design and Expert Analysis Software (version 13) generated factor combinations (PBD), polynomial equations, statistical outcomes, and other associated figures. Among eleven factors, discrete qualitative factors were *J* (mixing rate) and *L* (order of addition), and the rest were continuous quantitative factors.

### 2.3 Brunauer–Emmett–Teller (BET) Analysis

The surface area, pore radius, and pore volume of CaCO_3_ microparticles were calculated based on BET theory under STP (NOVAtouch LX gas sorption analyzers; Quantachrome, United States). Samples were degassed in a vacuum at a temperature of 120 °C for 3–4 h, and the adsorption isotherms were obtained using nitrogen as an adsorbate. The specific surface area, pore radius, and pore volume of the CaCO_3_ microparticles were evaluated by using the software tool integrated with the instrument.

### 2.4 Field-Emission Scanning Electron Microscopy (FESEM)

The surface morphologies of the prepared calcium carbonate particles were analyzed using FESEM (Carl Zeiss, Sigma 300) from Germany. To prepare samples for imaging, the dried and powdered samples were mounted onto brass stubs using double-sided tape and coated with gold in a vacuum. Imaging was performed at various resolutions and at an acceleration voltage of 5 kV to examine the surface features.

### 2.5 XRD Studies

The diffraction patterns of 16 calcium carbonate precipitates were analyzed using the Rigaku-Miniflex Powder XRD analyzer (Rigaku, Japan). The analyzer was equipped with a 30 kV generator and a 15 mA anode tube, and Cu Kα radiation was used to produce the diffraction patterns. The scanning range was set between 2θ values of 3–80° with a step size of 0.02° and a scanning rate of 2°/min, allowing for a comprehensive analysis.

### 2.6 FTIR Spectroscopic Studies

The FTIR spectra of 16 calcium carbonate precipitates were acquired by using the Shimadzu FTIR 8400S instrument manufactured by Shimadzu (Japan). The spectra were recorded within the range of 4000–600 cm^–1^, employing a resolution of 4 cm^–1^ and an accumulation of 35 scans. IR Solutions software was utilized to facilitate the analysis, encompassing various procedures such as background subtraction, baseline correction, normalization, spectrum recording, and other necessary calculations.

### 2.7 RS Studies

The Raman spectra of the 16 formulated calcium carbonate particles were collected using a Renishaw Raman inVia micro-Raman spectrophotometer. The instrument had 10× objectives, a notch filter to eliminate Rayleigh scattering, a monochromator, and a charge-coupled device (CCD) thermoelectrically cooled detector. Two light sources, an argon (Ar^+^) laser and a diode laser, were employed in the measurements. Three scans were averaged for each sample using WiRE software (version 3.3) to improve the signal-to-noise ratio. The obtained Raman spectra underwent baseline correction, and the Raman intensities were determined based on peak height measurements.

### 2.8 TGA

TGA was performed on the calcium carbonate particles. Initially, the samples were accurately weighed and placed in aluminum pans. The temperature was then incrementally increased from room temperature to 800 °C at a heating rate of 10 °C/min. The analysis was conducted under a continuous flow of dry nitrogen gas (50 cc/min) using a DTG 60 instrument manufactured by Shimadzu in Japan. The thermal stability of specimens was assessed by examining the degradation peaks and measuring the associated weight loss during the TGA analysis.

## 3 Results and Discussion

The conventional trial-and-error approach to screening critical input variables and developing a robust formulation with desired quality attributes remained a time- and energy-consuming approach until the advent of the experimental design concept. PBD is the most common screening design, which screens many factors and identifies critical ones with reasonable accuracy in a minimal number of runs.^11^ The input variables, their levels (three levels to elucidate the curvature effect), and the response outcomes are tabulated in Table 1.

**Table 1:** Input Variables, Their Levels (Three Levels to Elucidate the Curvature Effect), and the Response Outcomes as per the PBD^a^

code	A:pH	B: calcium chloride (M)	C: sodium carbonate (M)	D: temperature (celcius)	E: Gelucire 44/14 (w/v)	F: Cremophor RH40 (w/v)	G: Solutol HS15 (w/v)	H: Labrasol (w/v)	J: mixing rate	K: reaction time (h)	L: order of addition	surface area (m^2^/g)	pore radius (nm)	pore volume (cc/gm)
PB-1	4	1.0	2.0	4.0	4.0	4.0	1.0	4.0	slow	10.00	L1	4.5	2.97	6.70
PB-2	12	1.0	1.0	4.0	4.0	1.0	4.0	4.0	instant.	10.00	L2	9.48	6.68	31.68
PB-3	8	1.5	1.5	32.0	2.5	2.5	2.5	2.5	slow	5.04	L1	4.6	6.14	14.16
PB-4	12	2.0	1.0	4.0	1.0	4.0	1.0	4.0	slow	0.08	L2	16.14	4.7	37.97
PB-5	12	1.0	2.0	60.0	4.0	1.0	1.0	1.0	slow	0.08	L2	11.36	2.69	15.32
PB-6	4	2.0	2.0	4.0	4.0	4.0	4.0	1.0	instant.	0.08	L2	10.37	5.53	28.28
PB-7	12	1.0	2.0	60.0	1.0	4.0	4.0	4.0	instant.	0.08	L1	1.6	2.51	2.02
PB-8	12	2.0	1.0	60.0	4.0	4.0	1.0	1.0	instant.	10.00	L1	6.93	3.97	13.77
PB-9	8	1.5	1.5	32.0	2.5	2.5	2.5	2.5	instant.	5.04	L2	9.76	3.78	18.47
PB-10	4	2.0	1.0	60.0	4.0	1.0	4.0	4.0	slow	0.08	L1	2.47	4.49	5.56
PB-11	4	2.0	2.0	60.0	1.0	1.0	1.0	4.0	instant.	10.00	L2	6.87	4.53	15.58
PB-12	12	2.0	2.0	4.0	1.0	1.0	4.0	1.0	slow	10.00	L1	5.46	2.62	7.17
PB-13	8	1.5	1.5	32.0	2.5	2.5	2.5	2.5	slow	5.04	L2	7.86	4.01	15.79
PB-14	8	1.5	1.5	32.0	2.5	2.5	2.5	2.5	instant.	5.04	L1	5.43	5.93	16.12
PB-15	4	1.0	1.0	4.0	1.0	1.0	1.0	1.0	instant.	0.08	L1	1.5	3.22	2.43
PB-16	4	1.0	1.0	60.0	1.0	4.0	4.0	1.0	slow	10.00	L2	5.32	2.72	7.25

a L1: calcium chloride in the burette and sodium carbonate in the beaker; L2: sodium carbonate in the burette and calcium chloride in the beaker; slow: 2 mL/min; and instant: instantaneous.

The polynomial equations demonstrating the interplay between the critical factors and responses are given in Table 2.

**Table 2:** Polynomial Equations Demonstrate the Interplay between the Critical Factors and the Responses

coefficients	*R*1 (surface area)	coefficients	*R*2 (pore radius)	coefficients	*R*3 (pore volume)
Bo	+6.83	Bo	+5.02	Bo	+14.89
A-pH	**+2.05**	A-pH	–2.01	D-temperature	–4.56
*p*-value	<0.0001	*p*-value	<0.0001	*p*-value	0.050
L-order of addition	**+3.85**	C-sodium carbonate	+1.03	L-order of addition	+6.40
*p*-value	<0.0001	*p*-value	<0.001	*p*-value	0.0040
CK	+0.7954	E-Gelucire 44/14	–4.22		
*p*-value	<0.001	*p*-value	<0.0001		
HJ	+1.48	G-Solutol HS15	–1.95		
*p*-value	<0.0001	*p*-value	<0.0001		
KL	–1.12	K-reaction time	–3.25		
*p*-value	<0.0001	*p*-value	<0.0001		
		L-order of addition	–1.02		
		*p*-value	<0.0001		
		AC	–3.86		
		*p*-value	<0.0001		
		BL	–6.87		
		*p*-value	<0.0001		
		EG	+0.903		
		*p*-value	<0.001		
		AKL	+3.45		
		*p*-value	<0.0001		
model statistics					
model *p*-value	<0.0001	model *p*-value	<0.0001	model *p*-value	0.0045
*r*^2^	0.9972	*r*^2^	0.9936	*r*^2^	0.5690
adjusted *r*^2^	0.9948	adjusted *r*^2^	0.9807	adjusted *r*^2^	0.4971
model *f* value	420.32	model *f* value	77.35	model *f* value	8.41
curvature effect	significant (<0.0001)	curvature effect	not significant (>0.05)	curvature effect	not significant (>0.05)

Based on PBD, 16 formulations (PB-1–16) were prepared (Table 1), and the specific surface area, pore size, and pore volume were measured by nitrogen adsorption–desorption using the BET approach. The positive coefficient values in the final coded equation of specific surface area, pore radius, and pore volume suggested a synergistic effect, while negative coefficient values had an antagonistic effect (Table 2). Moreover, the residue formed through the PBD was subjected to characterization using XRD, FTIR, RS, and TGA techniques.

### 3.1 Influence of Independent Variables on Responses

**Figure 1:**
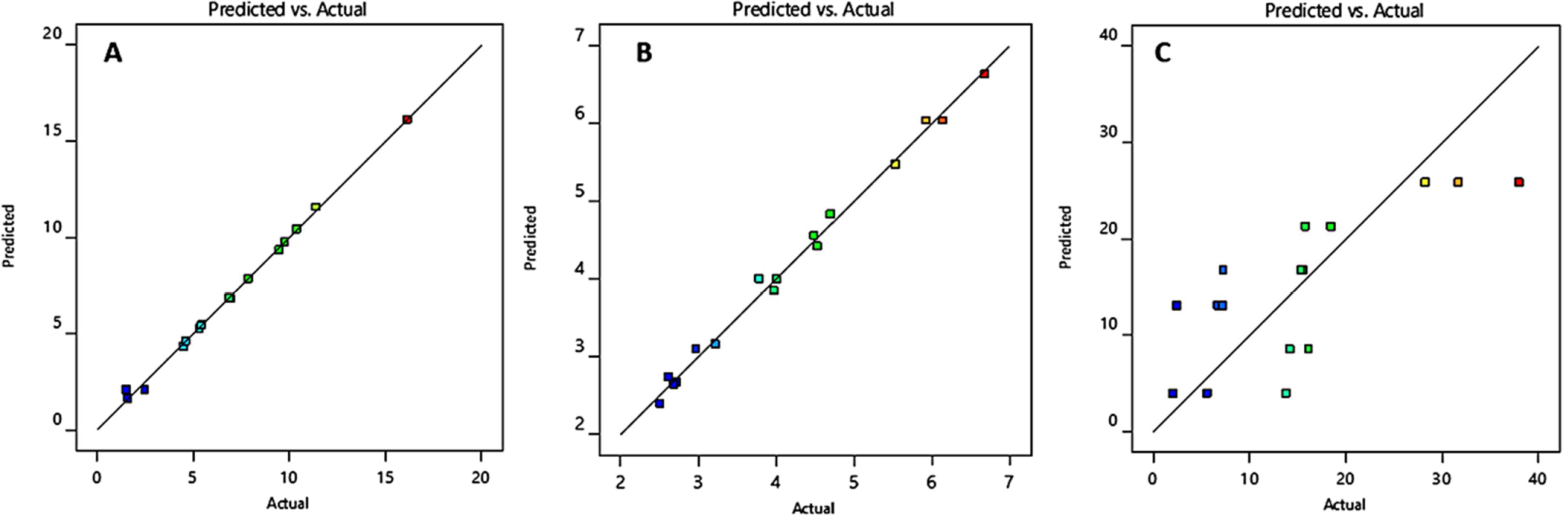
Correlation between predicted and actual response of the (A) surface area (*R*1), (B) pore radius (*R*2), and (C) pore volume.

The BET surface area (*R*1) of the prepared CaCO_3_ microparticles was found in the range of 1.5 m^2^/g (D15) to 16.14 m^2^/g (D4) (Table 1). The influential main factors identified were pH (A) (*p* < 0.0001) and order of addition (L) (*p* < 0.0001) (Table 2). The two factors’ interactions (2FI) that were significant in influencing the *R*1 were: (1) CK (molar concentration of sodium carbonate and reaction time, *p* < 0.001); (2) HJ (concentration of Labrasol-mixing rate, *p* < 0.0001); and KL (reaction time- order of addition, *p* < 0.0001). The reduced model carrying significant factors was found to be substantial (*p* < 0.0001), with a good correlation between *r*^2^ (0.9972) and adjusted *r*^2^ (0.9948) (Table 2), indicating an excellent correlation between the predicted and actual response of *R*1 (Figure 1A). The model *F* value was 420.32 with a significant curvature effect (*p* < 0.0001).

The pore radius (*R*2) of CaCO_3_ ranged from 2.62 nm (PB-12) to 6.68 nm (PB-2) (Table 1). The influential main factors for pore radius (*R*2) were: A (pH, <0.0001), C (molar concentration of sodium carbonate, <0.001), E (Gelucire 44/14, < 0.0001), G (Solutol HS15, <0.0001), K (reaction time, <0.0001), and L (order of addition, <0.0001) (Table 2). 2FI interactions affecting pore radius were (1) AC (pH and molar concentration of sodium carbonate, <0.0001), (2) BL (the molar concentration of calcium chloride and order of addition, <0.0001), and (3) EG (Gelucire 44/14 and Solutol HS15, < 0.001) (Table 2).

In this case, we have also observed a three-factor interaction between pH, reaction time, and order of addition (AKL, *p* < 0.0001) (Table 2). The reduced model carrying significant factors was found to be significant (*p* < 0.0001), with a good correlation between *r*^2^ (0.9936) and adjusted *r*^2^ (0.9807) (Table 2), indicating an excellent correlation between the predicted and actual response of *R*1 (Figure 1B). The model *F* value was 77.35 with a nonsignificant curvature effect (*p* > 0.005).

The response of the pore volume (*R*3) ranged from 2.43 cc/g (PB-15) to 37.97 cc/g (PB-4) (Table 1). Two main factors statistically influencing *R*3 were temperature (D, *p* = 0.050) and order of addition (L, *p* < 0.005) (Table 2). No higher-order interaction terms were found to influence pore volume. The reduced mathematical model carrying significant factors was found to be significant (*p* < 0.005), with a reasonable agreement between *r*^2^ (0.5690) and adjusted *r*^2^ (0.4971) (Table 2 and Figure 1C). The model *F* value was 8.41 with a nonsignificant curvature effect (*p* > 0.05). Since there was only moderate agreement between two *r*-squared values, we further investigated whether a power transformation was necessary for the response variable using the Box–Cox analysis (BCA) tool using Design and Expert analysis software.

**Figure 2:**
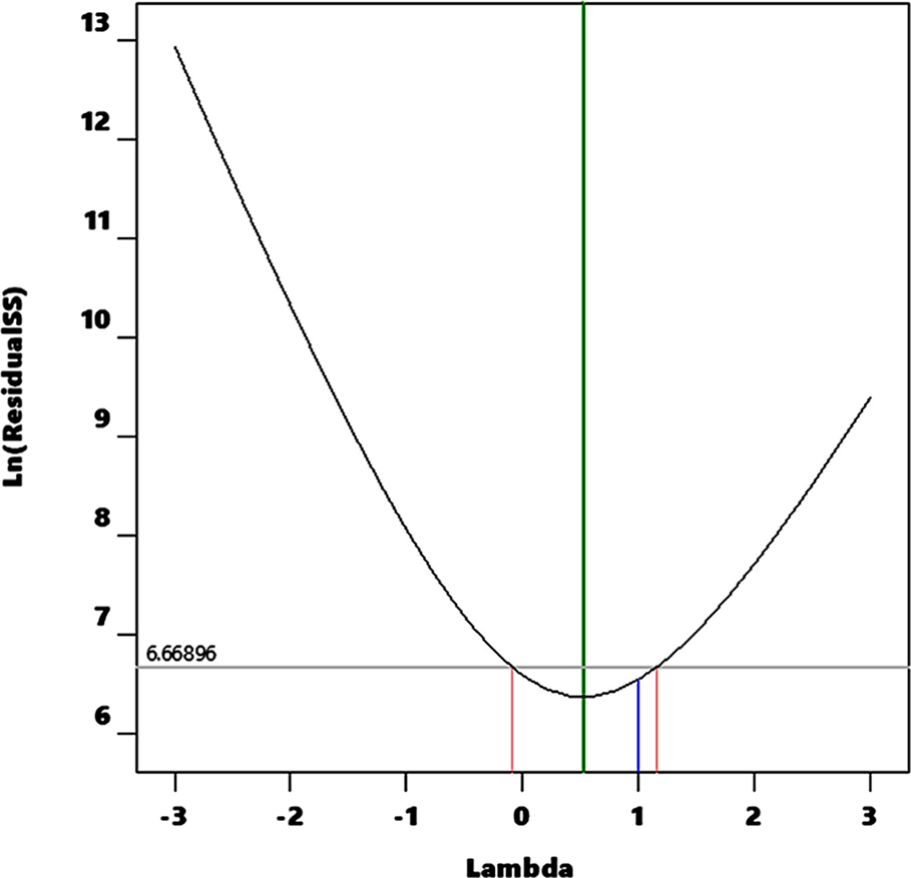
Box–Cox analysis of pore volume (*R*3) suggesting no specific transformation required to improve *r*^2^ (0.5690) and adjusted *r*^2^ (0.4971).

**Figure 3:**
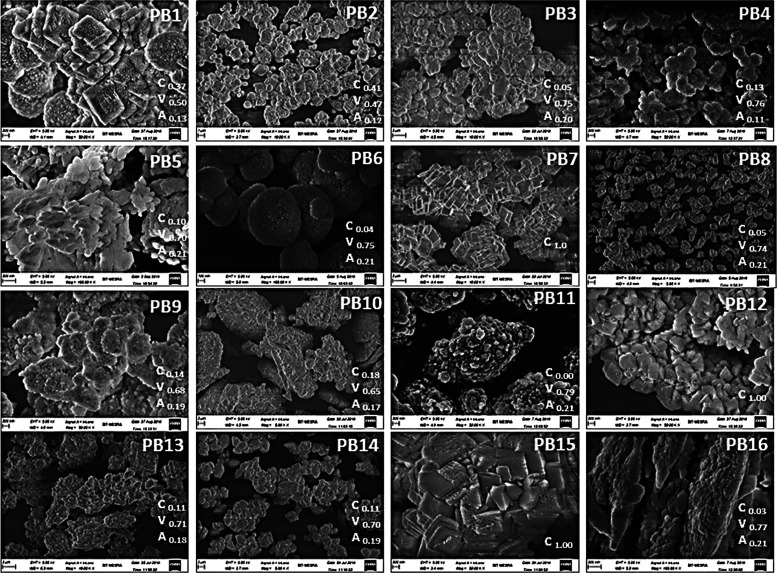
FESEM images of precipitates of calcium carbonate prepared as per the Placket–Burman design.

We used this method because the power law transformation applies only to positive ReV (as in this case). The plot (Figure 2) displays the currently applied lambda value (=1), the best-estimated lambda value (=0.52), and the confidence interval around the best-estimated lambda (−0.09, 1.15). Based on the plot, the software does not suggest a particular transformation if the confidence interval around the lambda value encompasses 1. The experimental results indicate that an increase in pH led to a linear increase in surface area for both L1 and L2 conditions. This suggests that the formation of vaterite particles is favored under these conditions. To provide further evidence for the formation of vaterite particles, the morphology of samples PB-4 (pH 12) (Figure 3) and PB-15 (pH 4.0) (Figure 3) were analyzed using FESEM (further discussed under FESEM studies).

The results confirm the formation of vaterite particles at high pH, as spherical mesoporous structures were observed, in contrast to calcite particles at low pH. In a previous report, the hexagonal vaterite disks formed when pH was increased to 10.^12^ Furthermore, in another study, spherical vaterite particles were produced within pH ranges of 8.0–9.5, while only pure calcite was made at low and high pHs.^13^ The surface area was higher under the L2 condition, in which the CO_3_^2–^ solution was added to the Ca^2+^ solution, compared to the L1 condition, in which the Ca^2+^ solution was added to the CO_3_^2^^–^ solution. This suggests that the order of addition (L) plays a role in the surface area of the CaCO_3_ particles. Additionally, as expected, the pore radius, which measures the size of the pores within the CaCO_3_ particles, decreased with an increase in pH for both the L1 and L2 conditions. However, the pH did not affect the pore volume (a measure of the total amount of space within the CaCO_3_ particles) under these conditions. This suggests that while the pH may play a role in forming the CaCO_3_ particles, it does not affect the overall pore structure of the particles. As shown in Table 2, the highest surface area (*R*1) of CaCO_3_ was observed when the CO_3_^2^^–^ solution was added to the Ca^2+^ solution (L2).

Similarly, the smallest pore radius and largest pore volume were found in the L2 condition. This indicates that the order of addition (L) significantly impacts the properties of the CaCO_3_ particles and can greatly influence the particles’ surface area, pore radius, and pore volume. The mixing mode plays a role in forming CaCO_3_ particles, as reported in the literature.^9^ When the CO_3_^2^–^^ solution is added to the Ca^2+^ solution, smaller and narrowly distributed particles are formed, while the opposite results in larger and nonuniformly sized particles due to the high initial pH of the carbonate solution, leading to high supersaturation and many nuclei forming and growing into relatively larger particles by attracting more free Ca^2+^ ions, resulting in a loss of uniformity.^9^

**Figure 4:**
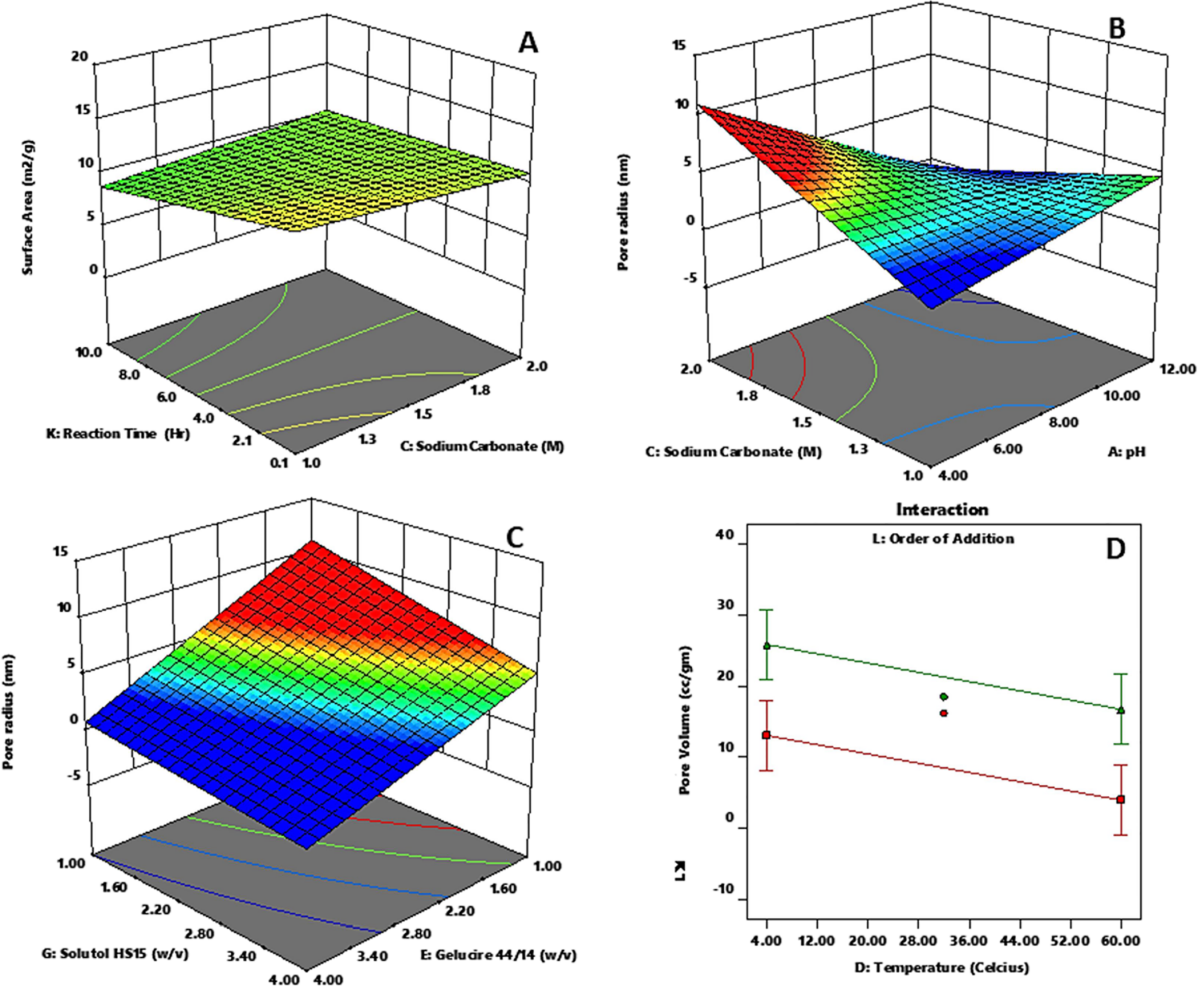
Response surface analysis of (A) surface area (m^2^/g) versus reaction time (h) and sodium carbonate (M), (B) pore radius (nm) versus sodium carbonate (M) and pH, (C) pore radius (nm) versus Solutol HS15 (w/v) and Gelucire 44/14 (w/v), and (D) pore volume (cc/g) versus temperature (celsius).

The results showed that the surface area (*R*1) is affected by the combined effect of the reaction time (*K*) and molar concentrations of sodium carbonate (C), as illustrated by a 3-D response curve (Figure 4A). Specifically, to achieve the maximum surface area of CaCO_3_ (hence vaterite formation), a lower reaction time (*K*) needs to be complemented with lower molar concentrations of sodium carbonate (C). Accordingly, the instantaneous mixing rate (*J*1) needs to be complemented with a lower Labrasol (H) to achieve maximum surface area. Additionally, if the content of Labrasol needs to be higher, a slow mixing rate must be used to achieve a larger surface area. Furthermore, reaction time should be kept minimum in the L2 system (when the CO_3_^2^–^^ solution was added to the Ca^2+^ solution) to achieve CaCO_3_ with maximum surface area.

The study found that the pore radius (*R*2) of the calcium carbonate crystals is affected by multiple factors, including pH, molar concentrations of sodium carbonate, the presence of certain surfactants (Gelucire 44/14 and Solutol HS15), reaction time, and specific reaction conditions (L2). It was found that as pH increases, pore radius decreases, and the same is true for an increase in Gelucire 44/14 content and Solutol HS15 content and an increase in reaction time (Table 2). Additionally, it was found that for a lower pore radius, higher molar concentrations of sodium carbonate should be used in conjunction with higher pH (Figure 4B), and higher molar concentrations of calcium chloride should be used in conjunction with the L2 condition. The study also indicated that the content of Gelucire 44/14 should be at the higher end within the explored factor levels of Solutol HS15 to achieve a lower pore radius (Figure 4C). Surfactants can affect the nucleation, growth, and polymorphism of calcium carbonate crystals by acting as nucleation sites, modifying the kinetics, and selectively promoting the formation of specific polymorphs. The presence of surfactant can affect the shape and type of CaCO_3_ particles. Studies have shown that surfactants like sodium dodecylbenzene sulfonate can change the crystals’ shape and promote the formation of unstable vaterite at high concentrations.^14^ In a study, the transition of CaCO_3_ crystals from the vaterite polymorph to aragonite was observed as the ratio of hexadecyl(trimethyl)azanium bromide to sodium dodecyl sulfate increased in a solution with a constant concentration of sodium dodecyl sulfate.^15^

**Figure 5:**
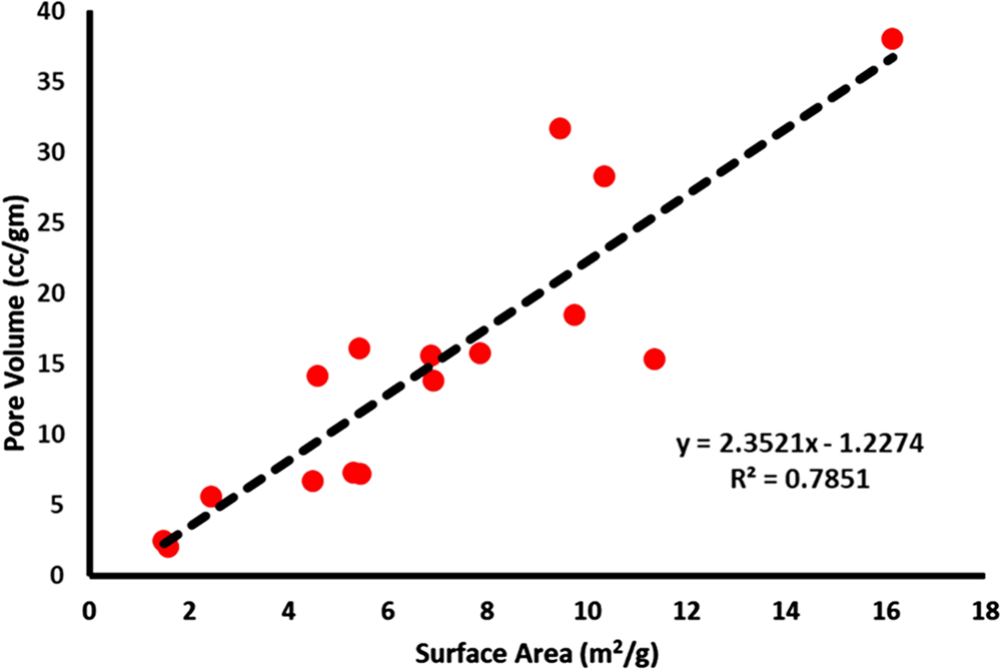
Correlation between the pore volume (cc/g) and surface area (m^2^/g).

Pore volume (*R*3) was maximum when the reaction was carried out at a lower temperature and under L2 conditions (Table 2 and Figure 4D). To further illustrate the relationship between surface area (*R*1) and pore volume (*R*3), the experimental values of both the responses (*R*1 and *R*3) were plotted on a rectilinear scale, and a nearly linear relationship was established with *r*^2^= 0.7851 (*y* = 2.3521*x –* 1.2274, *y*: pore volume; *x*: surface area) (Figure 5). A study found that increasing the reaction temperature to 80 °C led to the formation of calcite crystals with a less well-defined rhombohedral shape and secondary crystals on the surface.^16^ Other studies have also found that higher temperatures are more favorable for forming monodispersed cubic calcite particles.^17,18^ However, in a study, lower temperatures were found to be more effective for forming hollow CaCO_3_ microspheres, while higher temperatures resulted in nonhollow microspheres and irregularly shaped products.^19^

Previous studies have shown that monitoring the crystallization process over time can reveal necessary information about the formation of vaterite. For example, one study found that vaterite formation occurred immediately after combining the reactants and was visible after just 5 min of reaction time.^20^ Another study observed that hollow hexagonal vaterite disks were formed when the reaction time was increased.^12^ In a study, as the reaction time increased, the flower and hexagonal-shaped vaterite concentrations decreased, while the rod- and cluster-shaped aragonite concentrations increased up to 100% after 48 h.^9,15^ This is accompanied by an increase in the particle size.

Furthermore, the reaction time significantly impacted the crystallization behavior of the calcium carbonate polymorphs. The reaction time plays a crucial role in determining the amount and polymorphism of calcium carbonate formed, with different polymorphs having different nucleation and growth rates and the outcome being influenced by multiple parameters, including temperature, pH, and the presence of impurities or additives.^9^ A study reported that a short reaction time was favorable for the formation of the amorphous calcium carbonate (ACC) phase. In contrast, an increase in reaction time promotes the transformation of the unstable amorphous calcium carbonate phase into the thermodynamically stable calcite form.^21^ Additionally, when the reaction time was increased in different time intervals, it was found that a mixture of vaterite and aragonite was formed at reaction times of 6 and 12 h.^15^ An increase in reaction time to 24 h resulted in synthesizing a mixture of vaterite and aragonite, and extending the reaction time to 48 h resulted in the production of pure aragonite.^4^

An extensive analysis was carried out on the precipitates synthesized according to the PBD to support further the formed polymorphs’ identification. This analysis involved the utilization of various techniques, including FESEM, XRD, FTIR, RS, and TGA. The combined results from these characterization studies shall contribute to a more comprehensive understanding of the crystallinity, molecular structure, and thermal stability of the prepared CaCO_3_ precipitates.

### 3.2 FESEM Studies

The current study examined the FESEM images of 16 residues of CaCO_3_ using the PBD, as presented in Figure 3. CaCO_3_ polymorphs, such as calcite, aragonite, and vaterite, can be identified by their distinct morphologies under FESEM imaging, with calcite appearing as rhombohedral shapes with well-defined edges and smooth surfaces, aragonite as needle-like or acicular shapes with rough surfaces, and vaterite as spherical or cuboid shapes with the porous or rough surface.^5^ The images also indicate the relative mole fractions of calcite, vaterite, and aragonite (determined using an XRD study, as discussed in the section XRD Analysis). The factors contributing to a vaterite fraction exceeding 75% (Group 1) consisted of PB-3, PB-4, PB-6, PB-11, and PB-16 (Figure 3). Vaterite particles were observed to possess porosity and displayed diverse shapes, including spherical forms (PB-3, PB-4, and PB-6) and irregular shapes (PB-11 and PB-16). Likewise, a combination of factors resulted in a vaterite fraction ranging from 45 to 70% (Group 2), namely PB-1, PB-2, PB-5, PB-9, PB-10, PB-8, PB-13, and PB-14 (Figure 3). These factors exhibited various shapes of residues, such as spherical (PB-1, PB-2, PB-9, PB-10, and PB-14), elongated (PB-5), elliptical PB-8), and irregular (PB-13) (Figure 3). On the other hand, PB-7, PB-12, and PB-15 exclusively displayed calcite polymorphs (Group 3) with a cubical shape, and their composition was 100% calcite. The surface of PB-7 was characterized by smoothness, while that of PB-12 and PB-15 exhibited surface imperfections with minor pores (Figure 3).

### 3.3 XRD Analysis

**Figure 6:**
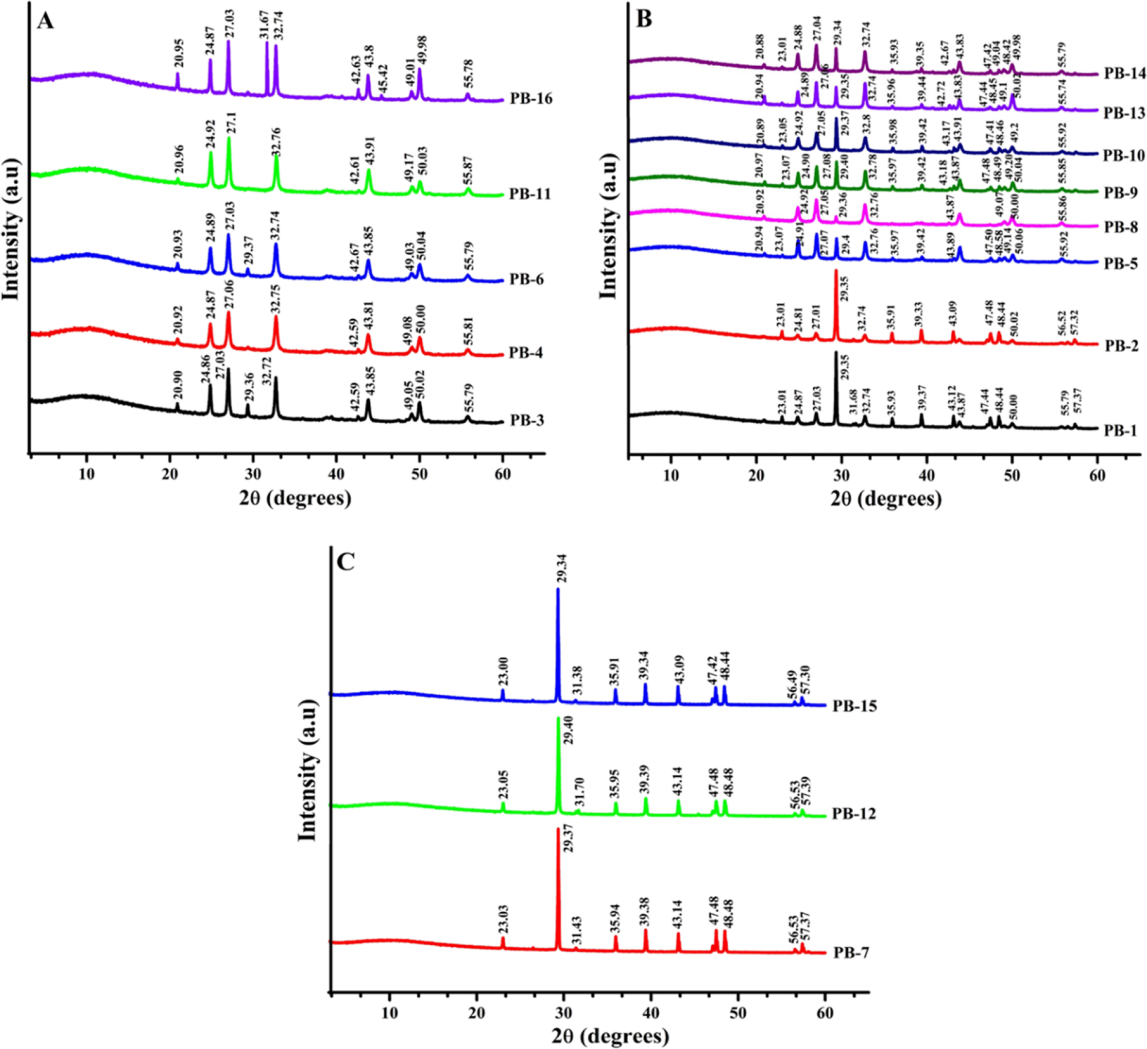
X-ray diffraction of calcium carbonate precipitates of Group 1 (A), Group 2 (B), and Group 3 (C) were prepared as per the PBD.

Figure 6A depicts the XRD pattern of calcium carbonate sediments with specific formulations, namely, PB-3, PB-4, PB-6, PB-11, and PB-16. These formulations belong to Group 1 and are characterized by a significant presence of vaterite forms, constituting more than 75% of the composition. By analyzing the XRD pattern, valuable information can be obtained regarding the crystallographic structure and arrangement of these residues. Similarly, Figure 6B showcases the XRD pattern of calcium carbonate precipitates (PB-1, PB-2, PB-5, PB-8, PB-9, PB-10, PB-13, and PB-14) within Group 2. These precipitates possess a formulation containing vaterite fractions ranging from 45% to 70%. The XRD pattern provides insights into the diffraction peaks and patterns associated with these specific compositions. In addition, Figure 6C presents the XRD stacks of PB-7, PB-12, and PB-15, which fall under Group 3. These residues are composed entirely of calcite forms, accounting for 100% of their composition.

The XRD stacks offer a comprehensive view of the crystallographic properties and diffraction behavior unique to calcite. These results were obtained using the Kontoyannis and Vagenas method,^22^ which analyzes the relative molar fractions of different polymorphs within Group 2 and Group 3. The molar fractions of calcite (XC), vaterite (XV), and aragonite (XA) were determined using eqs 1–3, respectively, as outlined below:
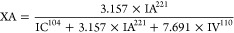
1
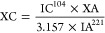
2
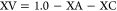
3

The aragonite, calcite, and vaterite mole fractions are indicated by XA, XC, and XV, respectively. The intensity peaks at 221, 104, and 110 reflection peaks for aragonite, calcite, and vaterite, respectively, are represented as IA^221^, IC^104^, and IV^110^. This method has been successfully utilized by us^5^ and other researchers^23,24^ to accurately determine the mole fractions of these mineral phases.

In the residues of Group 1, the highest percentage mole fraction was attributed to vaterite, ranging from 75.00 to 79.50%, followed by aragonite with a fraction of 19.8–20.00%, and calcite with a range of 0–13%. Most Group 1 precipitates were prepared under low-temperature conditions (4 °C) and at a low pH (pH 4). The temperature was considered a significant factor influencing the crystal morphology of the precipitated calcium carbonate polymorphs. Lower temperatures favored the formation of hollow microspheres.^25^ Cubic-shaped crystals were generally formed at room temperature, transforming into needle-like or stick-shaped structures at higher temperatures. Lower temperatures also facilitated the growth of smaller particles due to a higher nucleation rate, while higher temperatures resulted in the development of larger-sized particles. It was observed that most of the Group 1 (PB-4, PB-6, PB-11, and PB-16) followed the L2 order of addition, where sodium carbonate was added to the calcium chloride solution. This order of addition promoted the growth of spherical particles. Notably, calcite, the most stable polymorphic form, tends to transform from vaterite/aragonite to calcite. However, the excipients in Group 2 prevented this conversion to the calcite form.

In the sediments of Group 2 (PB-1, PB-2, PB-5, PB-8, PB-9, PB-10, PB-13, and PB-14), the highest percentage mole fraction was attributed to vaterite, ranging from 45 to 70%, followed by aragonite with a fraction of 12–20%, and calcite with a range of 5–41%. PB-1 contained calcite and vaterite polymorphs, with a molar fraction of 49% vaterite, 36% calcite, and a smaller amount of aragonite (13%). Similarly, PB-2 contained 46% vaterite and 41% calcite, as observed in the FESEM analysis (discussed in the FESEM studies). These findings demonstrate that various factors, such as temperature, pH, order of addition, and excipients, play a crucial role in determining the polymorphic composition and crystal morphology of precipitated calcium carbonate. Understanding these factors can facilitate control and customization of the properties of calcium carbonate materials for diverse applications in fields such as materials science, pharmaceuticals, and environmental remediation.

The sediments in Group 3 exhibited characteristic properties solely associated with calcite, without any traces of vaterite or aragonite. This aligns with previous studies that have yielded similar results. For instance, a study focusing on aqueous solutions of Ethylene Glycol observed only the characteristic peaks of calcite in the XRD results of precipitated CaCO_3_.^6^ Another study indicated that when precipitated CaCO_3_ was present in 100 g/L of Polyvinylpyrrolidone (PVP), it exhibited the characteristic peak of calcite.^8,26^ Furthermore, typical XRD patterns of precipitated CaCO_3_ using various fabricated Pluronic-surfactant templates revealed the crystal nature of calcite.^9^ The categorization of CaCO_3_ crystals into three distinct groups offers multiple potential applications. Group 1: vaterite-rich composition is ideal for drug delivery and biomedical applications. Group 2: mixed composition can be valuable in biocement and water treatment. Group 3: calcite forms have diverse applications in agriculture, industry, and optics.

### 3.4 FTIR Spectroscopy Studies

**Figure 7:**
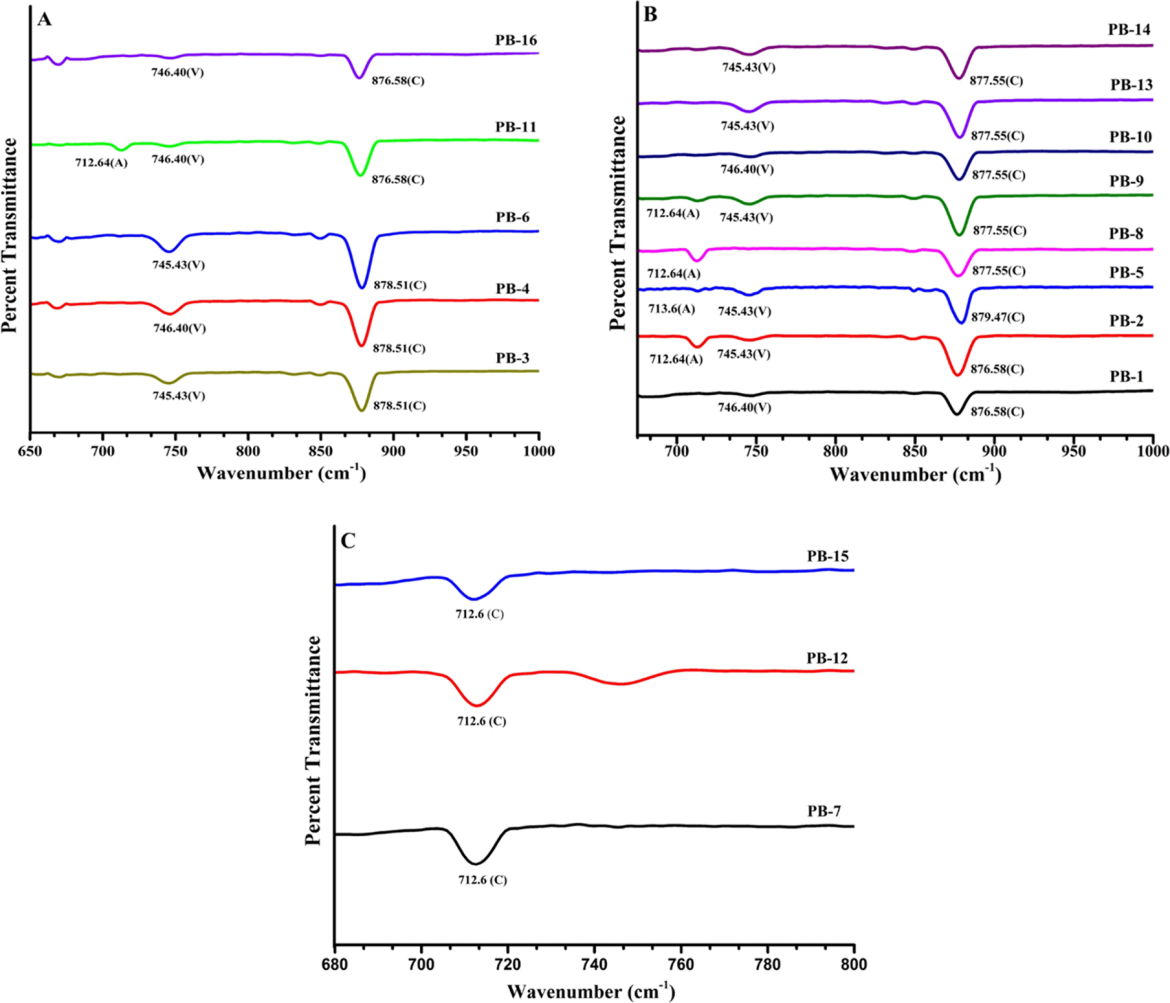
FTIR spectra of calcium carbonate precipitates of Group 1 (A), Group 2 (B), and Group 3 (C) were prepared as per the PBD.

The FTIR stacks in Group 1 (PB-3, PB-4, PB-6, PB-11, and PB-16) showed an intense band of calcite at 878.51 cm^–1^ (PB-3, PB-4, and PB-6) and 876.58 cm^–1^ (PB-11 and PB-16) (Figure 7A). This is in agreement with the reported calcite band at 876 cm^–1^ which happens due to asymmetric CO stretching mode (*n*_3_), CO_3_ out-of-plane formation (*n*_2_), and OCO in-plane deformation.^27,28^ The vaterite band is 746 cm^–1^ due to CO stretching and in-plane bending. It is present in all the formulations at 745.43 cm^–1^ (PB-3, PB-6) and 746.40 cm^–1^ (PB-4, PB-11, and PB-16).^28^ In the case of Group 2 (PB-1, PB-2, PB-5, PB-8, PB-9, PB-10, PB-13, and PB-14) (Figure 7B), the absorption bands of calcite are at 876.58 cm^–1^ (PB-1, PB-2) and at 877.55 cm^–1^ (PB-8, PB-9, PB-10, PB-13, and PB-14) which is similar to the data reported in the literature above.^28^ The vaterite band appears at 745.43 cm^–1^ in all cases and at 746.40 cm^–1^ in PB-1 and PB-10. Some other smaller peaks were visible in PB-2, PB-8, and PB-9 at 712.64 and 713.6 cm^–1^ in PB-5 due to the small amount of aragonite form present. Also, it is reported that the different morphologies of aragonite crystals display vibration bands at 712 and 713 cm^–1^.

The flake-like aragonite crystal shows a band at 713 cm^–1^ and the needle-like crystal at 712 cm^–1^ due to in-plane bending vibration (v_4_ cm^–1^).^29^Figure 7C shows the FTIR stack showcasing the calcium carbonate precipitates from Group 3 (PB-7, PB-12, and PB-15). The peak at 712.6 cm^–1^ corresponds to calcite, and no other peaks related to aragonite or vaterite were detected. This observation confirms the purity of the residues in the calcite form, which is consistent with the other findings.

### 3.5 Raman Spectroscopic Studies

**Figure 8:**
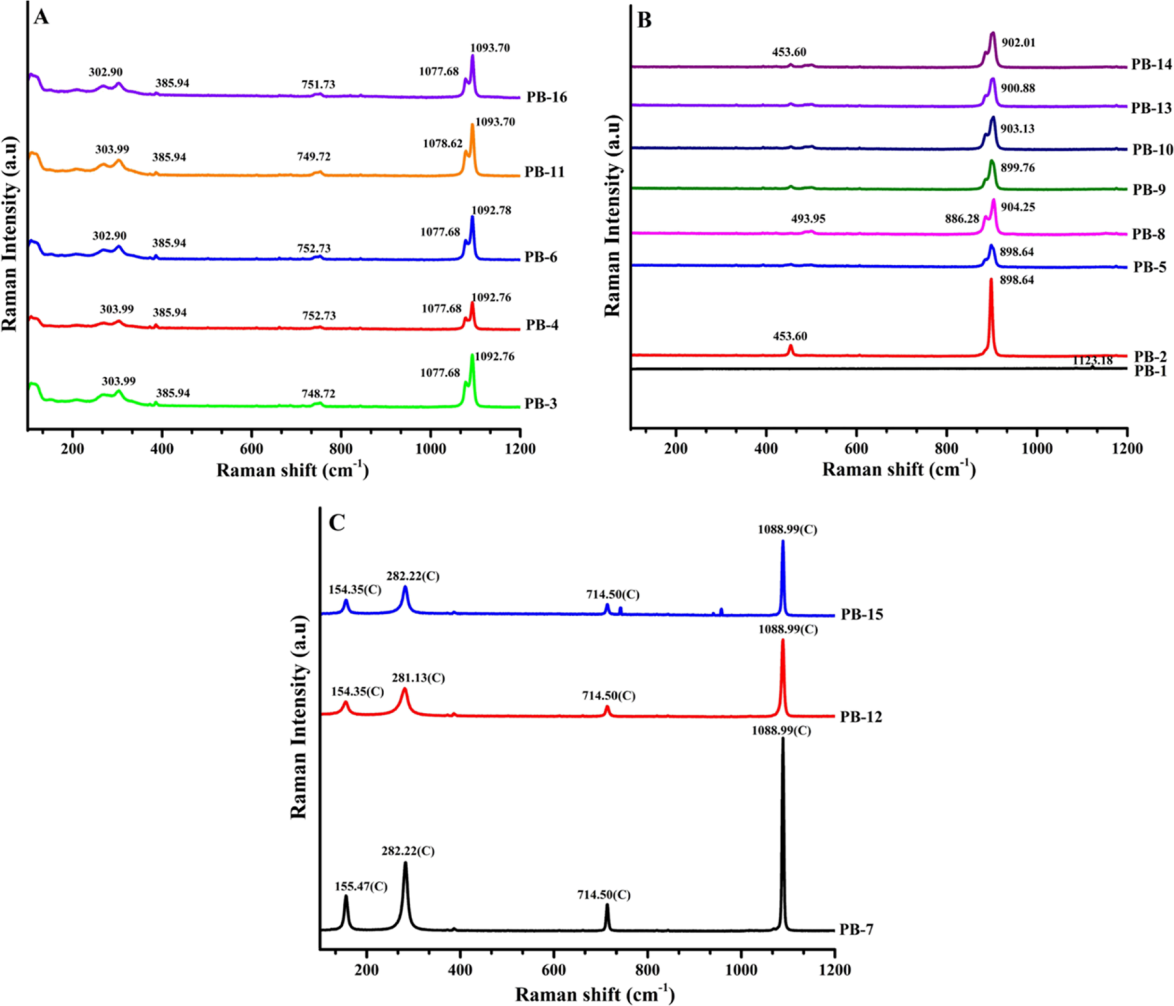
Raman spectra of calcium carbonate precipitates of Group 1 (A), Group 2 (B), and Group 3 (C) were prepared as per the PBD.

The Raman spectra of the calcium carbonate precipitate belonging to Group 1 (PB-3, PB-4, PB-6, PB-11, and PB-16) are depicted in Figure 8A. Accordingly, the Raman spectra of PB-1, PB-2, PB-5, PB-8, PB-9, PB-10, PB-13, and PB-14 (Group 2), and PB-7, PB-12, and PB-15 (Group 3) are stacked in Figure 8(B,C), respectively. Group 1 shows an intense peak in a splitting manner at 1092.76 cm^–1^ (PB-3, PB-4, and PB-6), 1093.70 cm^–1^ (PB-11, PB-16), 1077.68 cm^–1^ (PB-3, PB-4, PB-6, and PB-16), and 1078.62 cm^–1^ (PB-11) which corresponds to vaterite.

The Raman spectrum of the vaterite polymorph exhibits a significant peak at 1074 and 1090 cm^–1^, corresponding to the internal vibration mode (v_1_). This peak signifies the presence of two distinct symmetric stretching modes of the CO_3_ ions within the vaterite unit cell.^30^ The other peak of vaterite is also visible in the spectrum at 748.12 cm^–1^ (PB-3), 752.73 cm^–1^ (PB-4, PB-6), 749.72 cm^–1^ (PB-11), and 751.73 cm^–1^ (PB-16) which conforms with the reported peaks of vaterite.^30^ Also, one peak at 385.94 is visible in all of the CaCO_3_ precipitates of Group 1, and the other smaller bands at 302.90 (PB-6, PB-16) and 303.99 (PB-3, PB-4, and PB-11). All these are due to vibrations of complete unit cells, known as lattice modes.^31^ In Group 2, the major peak is at 898.64 cm^–1^ (PB-2, PB-5), 886.28 cm^–1^, 904.25 cm^–1^ (PB-8), 899.76 cm^–1^ (PB-9), 903.13 cm^–1^ (PB-10), 900.88 cm^–1^ (PB-13), and 902.01 cm^–1^ (PB-14) which indicates the major portion of vaterite in it.^32^ Consistent with the XRD studies, the CaCO_3_ precipitates from Group 3 (PB-7, PB-12, and PB-15) exhibit the presence of the pure calcite polymorphic form, as evidenced by the prominent reference peak at 1088.9 cm^–1^. The other peaks were assigned as 154.35 cm^–1^ (PB-12, PB-15), 155.47 cm^–1^, (PB-7) cm^–1^, 281.13 cm^–1^ (PB-12), and 282.22 cm^–1^(PB-7, PB-15).

This indicates the presence of pure calcite form.^33^ The intense and sharp peak observed at 1088.99 cm^–1^ in calcite corresponds to the symmetric stretching mode (v_1_) of the CO_3_^2–^ ion. It is reported that this peak’s position depends on the crystal structure of the carbonate mineral.^34^ Other peaks at 154.35 cm^–1^ (PB-12, PB-15), 155.47 cm^–1^ (PB-7), 281.13 cm^–1^ (PB-12), and 282.22 cm^–1^ (PB-7, PB-15) arise due to the translatory oscillations of the CO_3_ groups.^35^

### 3.6 TGA

**Figure 9:**
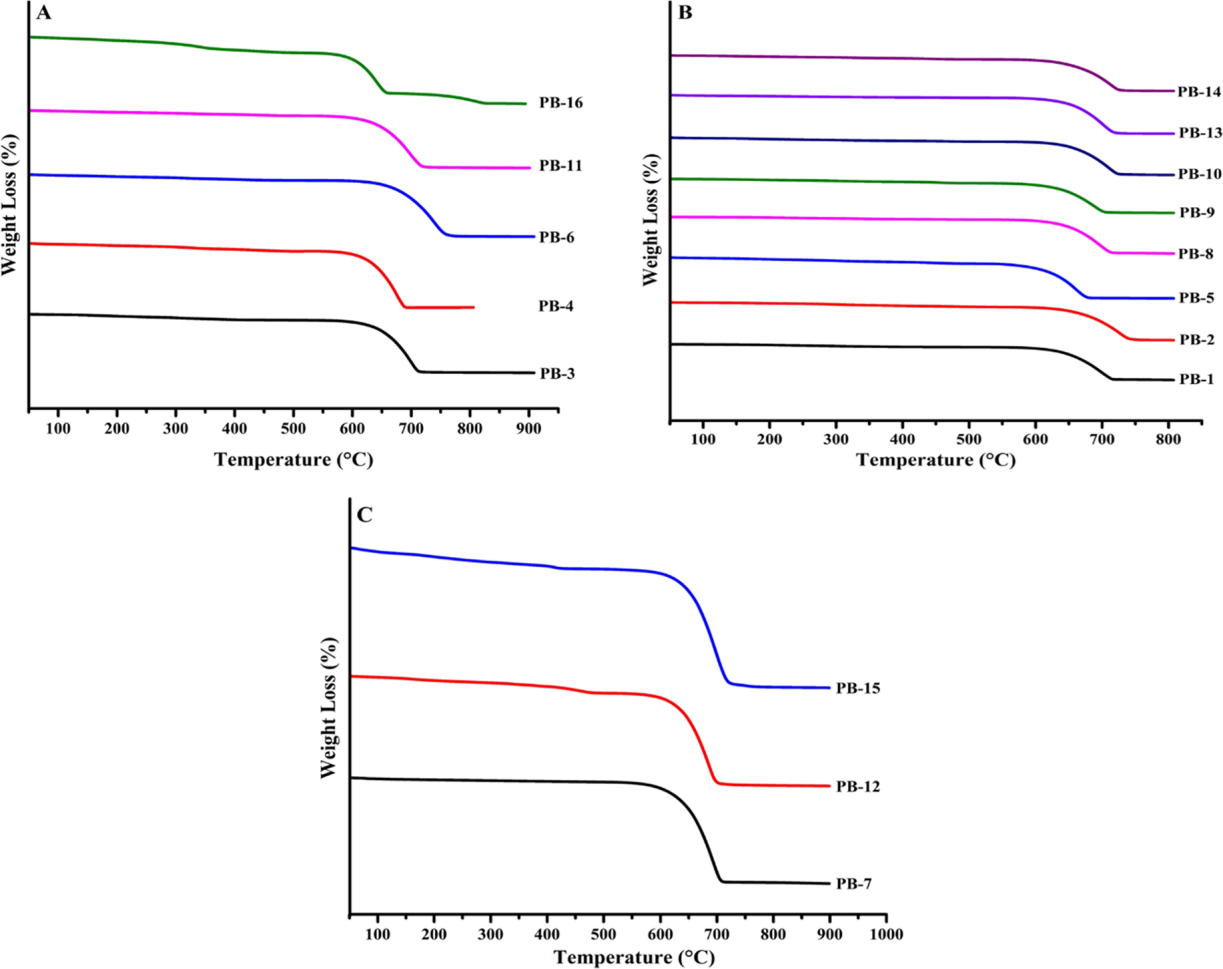
TGA thermogram of calcium carbonate precipitates of Group 1 (A), Group 2 (B), and Group 3 (C) prepared as per the PBD.

Figure 9(A–C) depicts the TGA thermograms of CaCO_3_ precipitates from different groups (Group 1 (Figure 9A), Group 2 (Figure 9B), and Group 3 (Figure 9C)). In the present study, PB-3 showed a total weight loss of approximately 40.59% (583.78–727.41 °C); PB-4 exhibited a weight loss of 44.62% (558.00–690.76 °C); PB-6 had a weight loss of 43.75% (598.08–771.23 °C); and PB-11 and PB-16 showed weight losses of 40.00 and 40.16% (594.15–732.01 °C).

Group 2 (Figure 9B, PB-1–2, PB-5, PB-8–10, and PB-13–14) exhibited a weight loss of 40–45.36%. The result can be summarized as: PB-1 (weight loss: 42.09%, 554.7–720.07 °C); PB-2 (weight loss: 42.65%, 577.66–747.31 °C); PB-8 (42.91%, 548.71–705.02 °C); PB-5 (44.88%, 534.23–698.03 °C); PB-9 (37.3%, 572–707.58 °C); PB-10 (42.08%, 584.24–721.95 °C); PB-13 (40.0%, 584.60–716.93 °C); and PB-14 (45.36%, 602.46–734.46 °C).

Similarly, the TGA curve of Group 3 (PB-7, PB-12, and PB-15) is shown in Figure 9C. PB-7 exhibited a weight loss of 41.36% (591–709 °C), while PB-12 had a weight loss of 38.72% (576.54–704.99 °C). Accordingly, PB-15 showed a weight loss of 48.00% (568.99–707 °C). These weight losses correspond to the major weight loss of CaCO_3_, indicating the decomposition of CaCO_3_ into CaO and CO_2_. The percentage weight mass loss of all precipitates falls within the approximately 40–50% range.

Similar findings were reported previously by Babou-Kammoe et al.,^36^ where they prepared CaCO_3_ particles with a peak temperature of TGA at 740 °C under controlled precipitation. Another study noted a DTG peak at 836.8 °C for pure CaCO_3_, which is associated with the main weight loss due to the transformation of CaCO_3_ into CaO. In another study, the DTG of pure CaCO_3_ showed a peak at 836.8 °C, corresponding to the major weight loss attributed to the transformation of CaCO_3_ into CaO.^37^

## 4 Conclusions

The purpose of our current research is to identify and assess critical independent variables that influence various porosity-related response variables, including surface area, pore radius, and pore volume. We utilized the PBD to screen and assess these variables. Additionally, we employed several instrumental techniques, namely, XRD, FTIR, RS, and TGA, to validate the type of precipitate formed through the PBD. The BET surface area (*R*1) of the prepared CaCO_3_ microparticles ranged from 1.5 to 16.14 m^2^/g. The pore radius (*R*2) of CaCO_3_ varied from 2.62 to 6.68 nm. The pore volume (*R*3) exhibited a range of 2.43–37.97 cc/g. We observed that the maximum pore volume (*R*3) was achieved at lower temperatures and under L2 conditions.

At high pH, we observed the formation of vaterite particles, which exhibited spherical mesoporous structures, while calcite particles formed at low pH. The order of addition impacted the surface area of the CaCO_3_ particles. However, under these conditions, the pH did not influence the pore volume, which measures the total amount of space within the CaCO_3_ particles. This indicates that while pH may play a role in forming CaCO_3_ particles, it does not affect the overall pore structure of the particles. To maximize the surface area of CaCO_3_ and promote vaterite formation, a lower reaction time should be combined with lower molar concentrations of sodium carbonate.

Similarly, a lower Labrasol concentration should be used with an instantaneous mixing rate to achieve a maximum surface area. The study revealed that the pore radius (*R*2) of calcium carbonate crystals is influenced by multiple factors, including pH, molar concentrations of sodium carbonate, the presence of surfactants such as Gelucire 44/14 and Solutol HS15, the reaction time, and specific reaction conditions. Lower pore radius values can be achieved by using higher molar concentrations of sodium carbonate in combination with a higher pH and higher molar concentrations of calcium chloride under L2 conditions. Furthermore, the study indicated that a higher content of Gelucire 44/14 within the explored factor levels of Solutol HS15 leads to a lower pore radius. To facilitate a comprehensive understanding of these instrumental techniques, we grouped the precipitates into three categories based on the percentage of vaterite formation: Group 1 comprises precipitates with vaterite percentages greater than 75%; Group 2 includes vaterite residues ranging from 45% to 70%; and Group 3 contains only calcite forms. The instrumental techniques successfully characterized the calcium carbonate precipitates and complemented each other in their analysis.
